# Employee Well-Being Profiles During COVID-19 Lockdown: A Latent Profile Analysis of French and UK Employees

**DOI:** 10.3389/fpsyg.2021.645300

**Published:** 2021-06-09

**Authors:** Lotta K. Harju, Joonas Rokka, Maíra Magalhães Lopes, Massimo Airoldi, Karine Raïes

**Affiliations:** Lifestyle Research Center, EMLYON Business School, Écully, France

**Keywords:** employee well-being, COVID-19, lockdown, latent profile analysis, multigroup CFA

## Abstract

The coronavirus pandemic, social distancing, and lockdown measures have had an impact on employee well-being. This study uses Latent Profile Analysis to examine subjective well-being among employees during the first lockdown based on a cross-national survey in UK and France (*n* = 652). We identify five distinct well-being profiles, namely Moderately positive (67%), Languishing (18%), Flourishing (8%), Mixed feelings (4%), and Apathetic (3%). The results showed that while some employees were suffering, others managed to thrive and cope with the stresses of the lockdown. We also found that the profiles could be distinguished by perceived changes in financial situation and physical health as well as experienced boredom. Our study complements prior studies that examine the relations between individual characteristics and well-being during the pandemic on a general level by showing that employee well-being under lockdown is not the same across the board.

## Introduction

This study examines well-being among French and UK employees during the first COVID-19 lockdown, when strict government-dictated social distancing and lockdown measures transformed the everyday life of people, not least in their working life. So far, research has mostly accounted for the vast negative impact of the pandemic and of the lockdown on health and subjective well-being (Jemberie et al., 2020; Patrick et al., 2020; Sibley et al., 2020). Research has also underlined the uneven well-being impacts—for example, in terms of gender, inequality, age, or low-income levels—and has suggested that a global virus outbreak or a similar type of socio-economic crisis tends to hit hardest those in a more vulnerable position (e.g., Enriquez and Goldstein, 2020; Etheridge and Spantig, 2020; Jaspal and Breakwell, 2020; Wang et al., 2020; Warren and Bordoloi, 2020; Collins et al., 2021).

Few studies focus on how the unfolding pandemic has affected employee well-being, and those have mainly reported on the strain experienced by specific vulnerable employee groups, such as frontline health care workers (Mackowiecki et al., 2020; Ripp et al., 2020; Saladino et al., 2020), blue-collar workers (Recchi et al., 2020), low-paid migrant workers (Wang et al., 2020), and increasingly precarious ‘gig economy’ workers, for example, those working in food delivery (Apouey et al., 2020). While these studies imply that less affluent workers are more at risk for poor health and well-being due to the pandemic, others indicate that higher socioeconomic status does not necessarily protect subjective well-being during lockdown. For example, a study conducted in the United States found that higher education was related to a greater increase in depressive symptoms and a decrease in life satisfaction during the pandemic (Wanberg et al., 2020). Studies also show that some precarious workers did not experience increased stress or anxiety during the first wave of the pandemic (Apouey et al., 2020). These contradictory findings suggest that employees differ in how the crisis impacts their well-being, and thus focusing on the average experience of the entire study population may oversimplify reality and hide important information concerning heterogeneity among employees (Hofmans et al., 2020).

The purpose of this paper is to address this gap and advance knowledge on employee well-being during lockdown by using Latent Profile Analysis (LPA) to identify different subpopulations of employees and by examining factors that distinguish these profiles. Distinctive from the more commonly employed variable-centered approach, LPA enables researchers to capture the heterogeneity of individuals in a group with respect to the phenomena under study and to understand how constructs combine within individuals (Marsh et al., 2009; Bennett et al., 2016; Spurk et al., 2020).

We seek to contribute to the literature on employee well-being under lockdown. Subjective well-being is often conceptualized as frequent positive experience and infrequent negative experience (Danna and Griffin, 1999). In this study, we adopt a broader concept of psychological well-being, which also comprises aspects related to positive functioning in life (Diener et al., 2009; Fisher, 2010; Huta and Waterman, 2014), such as positive relationships, positive self-perception, and a sense of purpose and competence in one's life (Ryff, 1989). This means that negative experiences alone do not jeopardize well-being, nor does having them exclude having positive experiences. We believe that this nuanced perspective enables us to advance a more comprehensive understanding on employee subjective well-being under lockdown, particularly because in such a context observing only strains and concerns provide a rather narrow lens on employee experiences.

In addition, our study contributes to the existing literature on employee well-being during the pandemic across countries (e.g., Bidzan-Bluma et al., 2020; Ebert et al., 2020; Gubler et al., 2020; Paredes et al., 2020; Risi et al., 2020; Sibley et al., 2020), by examining employees in France and the UK, two countries that were hit particularly hard in Europe, each counting more than 50,000 deaths caused by the coronavirus at the time of the study. A further strength of our study is that it was carried out during the strict lockdown period of eight weeks that both countries introduced in April/May 2020. Although studies have examined the pandemic's overall impact on well-being, they have missed providing evidence on the heterogeneous nature of employee well-being during the lockdown period.

Due to the exploratory nature of the study, we did not make any assumptions about the profiles or their prevalence, but rather attempted to answer the following research questions: (1) Can different profiles of well-being be identified among employees during (the first) lockdown, and if so, how prevalent are these profiles? (2) Can these profiles be distinguished by characteristics pertaining to the professional and private domains? and (3) Do socio-economical and work-related factors affect the likelihood of belonging to a certain profile during lockdown? In other words, our objective is to uncover whether and why some employees may thrive while others suffer under lockdown.

## Methods

### Participants

The data was collected via Qualtrics online participant pool and consisted of French and UK employees who were working or employed during the lockdown. The participants filled in the survey in May 2020, when lockdown measures had been in place for more than a month in both countries. After omitting 109 respondents based on checks of overly short response times (i.e., <5 min) and four respondents based on clear outlier patterns, the final sample of this study consisted of 652 employees residing in France (*N* = 326) and in the UK (*N* = 326).

Among the participants, 38% were working remotely (i.e., outside of workplace), 28% were in an employment contract but not working during the lockdown, and 27% reported being alone in lockdown. About half of the sample (53%) was female, 45% were under 35 years of age, 41% had a high school diploma, 36% held a bachelor's degree and 16% had a higher university degree. The participants worked as managers and highly skilled professionals (19%), clerks and administrative assistants (23%), skilled manual workers (14%), teachers, nurses or lower-level professionals (11%), service workers (8%), and specialized workers, technicians, and store managers (6%). For 23% of the participants, the amount of work did not change, while for 61% the amount of work decreased.

### Measures

Well-being was assessed with a three-dimensional instrument that captured positive and negative experiences as well as psychosocial well-being (PWB; Diener et al., 2009). Positive and negative feelings were assessed by asking the participants to reflect on the time during lockdown and to respond to how often they had experienced specific feelings. The response scale ranged from 1 (= never) to 7 (= always). Positive feelings were captured by six items describing pleasant experiences (e.g., “joyful,” “happy”), and negative feelings were captured by six items describing unpleasant experiences (e.g., “angry,” “sad”). Psychosocial well-being consisted of eight items that described the experience of one's social relationships, purposeful life and interest, and a sense of self-respect and competence (example item: “I lead a purposeful and meaningful life”). Responses were scaled from 1 (= strongly disagree) to 7 (= strongly agree).

We also added single-item measures assessing self-rated changes in workload in the professional and personal domains. Specifically, we asked participants to assess the extent to which there had been changes in the amount of work, household chores, and childcare responsibilities during the lockdown. The response scale for these three measures ranged from 1 (= considerably decreased) to 7 (= considerably increased). We also inquired about the extent of changes in the financial situation of the household during the lockdown (response scale from 1 = considerably worse to 7 = considerably better). Self-rated change in physical health was assessed with a single item adapted from McColl-Kennedy et al. (2017), i.e., To what extent has your physical health changed during the lockdown. The response scale ranged from 1 = considerably decreased to 7 = considerably increased. In addition, boredom was assessed with a single item describing how often the participants experienced boredom during lockdown (response scale ranged from 1 = never to 7 = always).

Socioeconomic and occupational factors were dichotomized and included age (0 ≤ 35years, 1 ≥ 35 years), country (0 = France, 1 = UK), gender (0 = Male, 1 = Female), education (0 = low level of education, 1 = Bachelor or higher university degree), and income (0 ≥ Euro 2,500/month, 1 ≤ Euro 2,500/month). Occupational factors included working remotely (0 = No, 1 = Yes) and having a managerial or professional position (0 = No, 1 = Yes).

### Analytical Procedure

All analyses were carried out using the MPlus statistical package (version 8; Muthèn, L. K and Muthèn, 1998–2017) and the full information maximum likelihood estimation (FIML) with standard errors robust to non-normality (MLR estimator). As a preliminary step, we conducted confirmatory factor analyses (CFA) on the measurement model consisting of three dimensions of subjective well-being (i.e., six items reflecting positive experiences, six items reflecting negative experiences, and eight items reflecting psychosocial well-being) among the French and British participants. Specifically, we tested measurement invariance across the countries to examine if the constructs were captured in a comparable way in the two countries [see Van de Schoot et al. (2012) for the procedure].

The fit of the model to the data was examined with the χ^2^ goodness-of-fit statistic, the Root Mean Square Error of Approximation (RMSEA), the Tucker-Lewis Index (TLI), the Comparative Fit Index (CFI), and the Standardized Root Mean Square Residual (SRMR). Generally, models with TLI and CFI > 0.95, and RMSEA and SRMR < 0.05 represent a very good fit between the hypothesized model and the data (Little, 2013). Because in larger samples, change in χ^2^ may not reliably reflect the significance of change, we employed a rigorous significance level of *p* < 0.001 and change in CFI (ΔCFI < 0.01; Cheung and Rensvold, 1999) to assess differences between models.

Thereafter, LPA was employed to investigate whether different subgroups of employees could be identified based on their mean levels of well-being indicators, i.e., the frequency of positive and negative experiences during lockdown and psychosocial well-being. LPA allows for empirical distinction of fairly homogenous groups of people within the sample, and unlike traditional clustering approaches, it takes into account the goodness-of-fit of the model and the measurement error (Morin et al., 2016). Thus, the best fitting model was chosen based on the variety of statistical indicators as well as the substantive meaning of the solution (Marsh et al., 2009).

We performed analyses with 1,000 random sets of start values with 200 iterations, and retained the 200 best solutions for final stage optimization (Hipp and Bauer, 2006). The decision of the number of latent classes was based on different criteria. Akaike Information Criterion, Bayesian Information Criterion (BIC), and the sample-adjusted BIC (aBIC) were used to assess model fit. Lower values indicated a better fit of the model. In addition, the bootstrap likelihood ratio test was employed to compare solutions with different numbers of latent classes (*k* or *k – 1* number of classes). A low *p* value (<0.05) indicated that the *k*−*1* model must be rejected in favor of a model with at least *k* classes.

For large samples, it is typical for aBIC to keep decreasing in favor of more profiles. For this reason, we assessed the profile solution using additional criteria recommended by Nylund et al. (2007) as well as Meyer and Morin (2016), specifically BIC and slowing down of model improvement. We also evaluated the profiles from a theoretical perspective to avoid over-interpretation of the empirical results (Lubke and Muthén, 2005; Morin et al., 2016).

The clarity and interpretability of the latent class solution was also assessed in deciding on the number of classes. For this purpose, the distinctiveness of the profiles was evaluated by assessing entropy values, which describe the accuracy of the overall classification, and average latent class posterior probabilities (AvPP), which assess the likelihood of an observation being assigned to a specific profile. For entropy, the closer the value is to 1 (from 0 to 1) the better the classification is (Celeux and Soromenho, 1996). For average latent class posterior probabilities, values higher than 0.70 indicate that the solution may be interpreted using the mean profiles (Nagin, 2005).

Next, we tested the significance of mean-level differences in profile characteristics across all specific pairs of profiles using the modified Bolck-Croon-Hagennars (BCH) approach (Bakk and Vermunt, 2016), which refers to a bias-adjusted modeling technique to evaluate means of continuous variables across latent profiles [see Asparouhov and Muthén (2014a) for further details of the procedure]. We also used the R3STEP procedure (Asparouhov and Muthén, 2014b), which is a three-step method for adding latent profile predictor variables. In R3STEP, a multinomial logistic regression is performed, wherein latent profile membership is regressed on selected covariates [see Asparouhov and Muthén (2014b) for further details]. We used this technique to assess the role of dichotomous socio-economical and occupational factors (i.e., age, female, university education, income, managerial or professional position, and teleworking) in predicting profile membership.

## Results

### Preliminary Analyses

Internal reliabilities of the measures (i.e., Cronbach's alpha), descriptive statistics and correlations between the study variables are presented in Table 1.

**Table 1:** Descriptive statistics of the study variables.

		***M***	***SD***	**α**	**1**	**2**	**3**	**4**	**5**	**6**	**7**	**8**	**9**
1	Positive experiences	4.2	1.03	0.87									
2	Negative experiences	3.8	1.19	0.86	−0.19^***^								
3	Psychosocial well-being	4.7	1.09	0.89	0.53^***^	−0.08^*^							
4	Perceived change in financial situation	3.3	1.31	NA	0.20^***^	−0.12^**^	0.20^***^						
5	Perceived change in physical health	3.9	1.40	NA	0.26^***^	0.03	0.26^***^	0.24^***^					
6	Perceived change in workload	2.9	1.73	NA	0.06	0.09^*^	0.11^**^	26^***^	0.09^*^				
7	Perceived change in household chores	4.7	1.67	NA	0.09^*^	0.07	0.16^***^	0.03	0.15^***^	0.14^***^			
8	Perceived change in childcare	4.7	1.68	NA	0.17^***^	0.00	0.11^**^	0.06	0.09^*^	−0.00	0.32^***^		
9	Boredom	4.2	1.64	NA	−0.17^***^	0.52^***^	−0.06	−0.10^*^	0.04	0.01	0.09^*^	0.04	
10	No. People in lockdown	2.6	1.38	NA	0.15^***^	0.07	0.09^*^	0.04	0.08^*^	0.03	0.06	0.16^**^	−0.01

*** *p < 0.001*,

** *p < 0.010*,

* *p < 0.05*.

The measurement model for the three-dimensional model of well-being across UK and France was specified by allowing each indicator to load on its respective latent factor without constraints (i.e., eight items reflecting psychosocial well-being, six items reflecting positive experiences, and six items reflecting negative experiences) in both sub-samples (i.e., configural model; ${\text{x}}_{\left(\text{df}\right)}^{2}$ = 774.379(334), CFI = 0.901, TLI = 0.888, RMSEA = 0.064; SRMR = 0.057). Modification indices suggested that the model fit could be improved by letting the residuals between two items reflecting negative experiences (“negative” and “unpleasant”) and two items reflecting positive experiences (“joyful” and “happy”) correlate in both sub-samples because of overlapping item content. The modified model showed an acceptable fit (${\text{x}}_{\left(\text{df}\right)}^{2}$ = 652.081(330), CFI = 0.928, TLI = 0.917, RMSEA = 0.055; SRMR = 0.057).

Next, we compared this model to one where factor loadings were constrained to be equal across the two samples to assess whether the meaning of the well-being constructs were the same across the groups [i.e., metric model; $x_{\left(\text{df}\right)}^{2}$ = 672.660(350), CFI = 0.928, TLI = 0.921, RMSEA = 0.053; SRMR = 0.069], and did not find statistically significant difference to the configural model [$\Delta x_{\left(\Delta \text{df}\right)}^{2}$ = 18.8191(20), *p* = 0.5336]. Finally, we compared the metric model to one where both factor loadings and intercepts were constrained across groups [i.e., scalar model; $x_{\left(\text{df}\right)}^{2}$ = 743.923(370), CFI = 0.916, TLI = 0.914, RMSEA = 0.056; SRMR = 0.071; $\Delta x_{\left(\Delta \text{df}\right)}^{2}$ = 82.5673(20), *p* < 0.001]. These tests meant that there was only partial measurement invariance across countries, which further investigation revealed was due to the intercepts of one item reflecting PWB (i.e., “people respect me”) and one item reflecting negative experiences (i.e., “angry”). The differences in these intercepts implied that the meanings of the levels of the underlying items were different across the countries. We followed the recommendation by Cheung and Rensvold (1999) and omitted these items in order to make reliable interpretations of the composite scores (Steinmetz, 2013). The resulting model was invariant across samples [${\text{x}}_{\left(\text{df}\right)}^{2}$ = 709.890(368), CFI = 0.923, TLI = 0.921, RMSEA = 0.053; SRMR = 0.069; $\Delta X_{\left(\Delta \text{df}\right)}^{2}$ = 38.1332 (18), *p* = 0.004].

### Main Analyses

As the correct number of latent profiles was not known a priori, we tested models for up to seven profiles (Table 2).

**Table 2:** Latent profile fit statistics.

**Number of profiles**	**LL**	**Free parameters**	**AIC**	**BIC**	**SABIC**	**BLRT**	**Entropy**	**Proportions for the profiles (%)**
1	−2964.338	6	5940.675	5967.556	5948.506			
2	−2889.203	10	5798.405	5843.206	5811.456	0.000	0.61	72/28
3	−2847.424	14	5722.848	5785.569	5741.119	0.000	0.73	72/16/12
4	−2819.671	18	5675.342	5755.983	5698.833	0.000	0.74	28/2/9/61
5	−2783.607	22	5611.215	5709.776	5639.926	0.000	0.81	3/18/4/67/8
6	−2770.993	26	5593.986	5710.467	5627.917	0.000	0.72	38/3/6/42/3/8
7	−2759.182	30	5578.364	5712.766	5617.516	0.000	0.78	3/15/1/11/13/ 4/53

*LL, log likelihood; AIC, Akaike Information Criterion; BIC, Bayesian Information Criterion; SABIC, sample-adjusted BIC; BLRT, bootstrap likelihood ratio test*.

**Figure 1:**
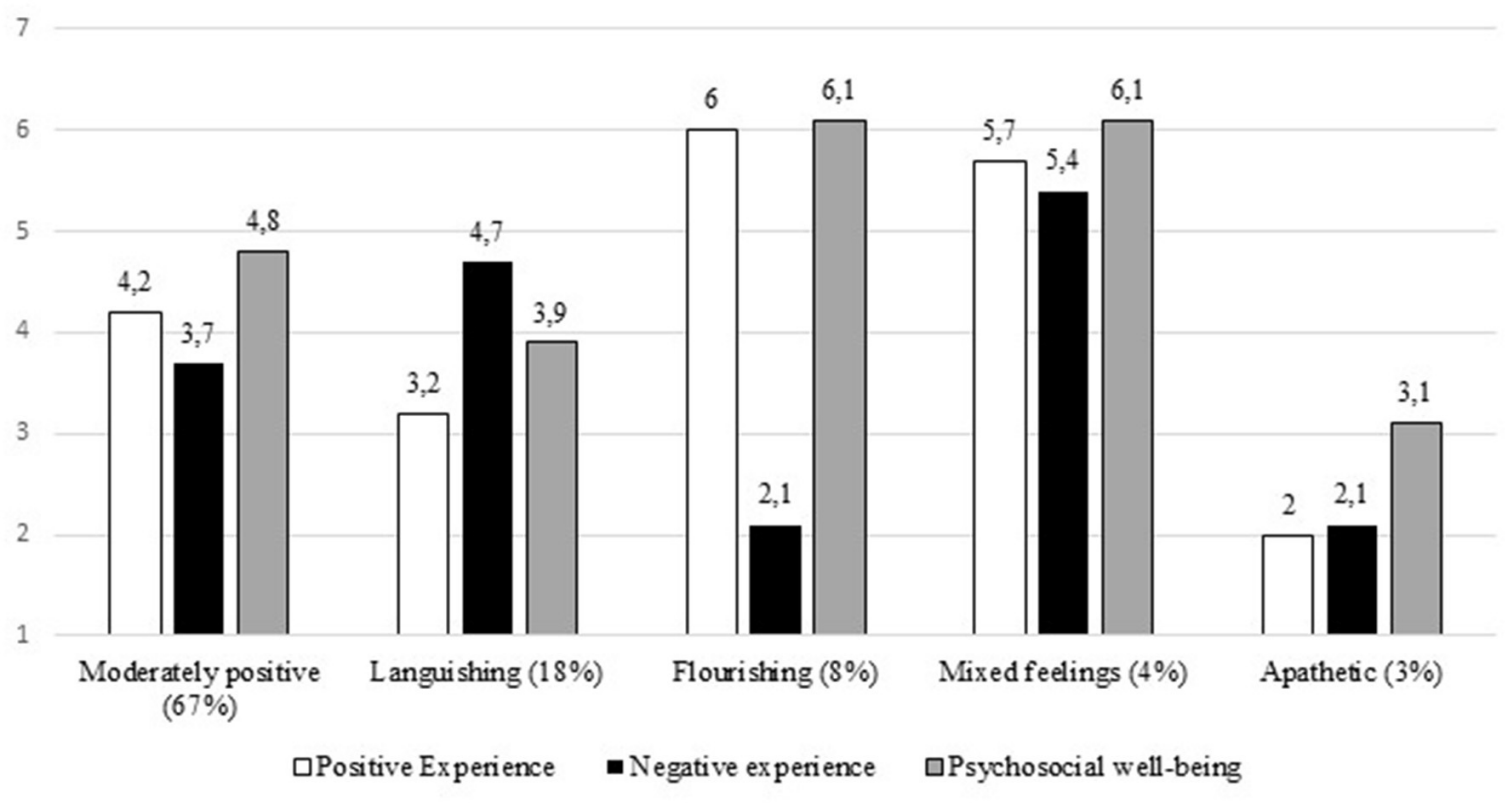
Well-being profiles during lockdown (*N* = 652). Mean scores for each well-being dimension across well-being profiles are represented above bars.

The fit criteria implied that model improvement slowed down at five profiles and the quality of the profiles decreased after adding the sixth profile, which are indicators of an inferior profile solution (Nylund et al., 2007; Meyer and Morin, 2016). After assessing the clarity and interpretability of the profiles, a five-profile solution was chosen. Entropy criterion (0.81) and the AvPP for profile membership (range 0.72–0.94) supported the quality of the profile solution. As an answer to our first research question, five distinct employee well-being profiles were identified, presented in Figure 1.

Addressing our second research question we found differences across the profiles particularly in the number of people in lockdown, changes in financial situation, changes in physical health, and experiences of boredom. Table 3 shows the means of all the characteristics across profiles. Our third research question concerned the associations between the socio-economical and occupational covariates and profile membership. Results did not show statistically significant effects of the covariates (i.e., country, age, gender, income, education, occupational status, and telework) in predicting profile membership (see Table 4 in Supplementary Material). We will next introduce the employee well-being profiles and their characteristics during lockdown.

**Table 3:** Differences in “lockdown characteristics” across profiles (*N* = 652).

**Profile (% of the sample)**	**No of people in lockdown *M(SE)***	**Equality test of means (χ^2^)**	**Change in amount of childcare *M(SE)***	**Equality test of means (χ^2^)**	**Change in household chores *M(SE)***	**Equality test of means (χ^2^)**	**Change in workload *M(SE)***	**Equality test of means compared to the average (χ^2^)**	**Change in financial situation *M(SE)***	**Equality test of means compared to the average (χ^2^)**	**Change in physical health *M(SE)***	**Equality test of means compared to the average (χ^2^)**	**Experience of boredom *M(SE)***	**Equality test of means compared to the average (χ^2^)**
Moderately positive (67%)	2.7 (0.08)		4.8 (0.10)		4.8 (0.09)		2.9 (0.09)		3.3 (0.07)		4.0 (0.07)		4.1 (0.08)	
Languishing (18%)	2.4 (0.18)	1.360	4.3 (0.28)	3.214	4.4 (0.25)	1.856	2.7 (0.20)	0.859	2.7 (0.16)	12.505^***^	3.1 (0.18)	16.226 ^***^	5.3 (0.20)	25.483^***^
Flourishing (8%)	2.8 (0.20)	0.215	4.9 (0.34)	0.014	4.9 (0.35)	0.082	2.9 (0.28)	0.004	4.0 (0.24)	7.227^**^	4.4 (0.24)	2.740	2.2 (0.23)	60.922^***^
Mixed feelings (4%)	3.3 (0.28)	4.436^*^	4.1 (0.42)	2.941	4.4 (0.43)	0.749	3.8 (0.48)	2.968	3.9 (0.41)	2.016	5.2 (0.34)	12.915 ^***^	5.4 (0.39)	8.824^**^
Apathetic (3%)	2.1 (0.28)	4.229^*^	3.5 (0.46)	7.723^**^	3.4 (0.56)	5.877^*^	3.0 (0.55)	0.048	3.1 (0.36)	0.368	2.7 (0.37)	10.785 ^***^	3.0 (0.49)	5.736^*^

*** *p < 0.001*,

** *p < 0.010*,

* *p < 0.05*.

*Means of each profile are compared to the means of the Moderately positive profile, which is considered as the normative profile. Statistically (non-) significant difference thus indicates that the mean of a given characteristic in the target profile is (not) statistically significantly different from that of the comparison profile (i.e., the Moderately positive)*.

### Moderately Positive Well-Being Profile

The “Moderately positive” profile involves those participants that reported occasional positive (*M* = 4.2) and negative (*M* = 3.7) experiences and somewhat positive psychosocial well-being (*M* = 4.8) during the lockdown. This profile was the largest as it contained 67% of the participants. Hence, we consider this as the normative profile to which other profiles will be compared. Participants with this profile were in lockdown with more than one other person (*M* = 2.7), reported moderate increase in childcare responsibilities (*M* = 4.8) and household chores (*M* = 4.8), and a moderate decrease in workload (*M* = 2.9) and the financial situation of the household (*M* = 3.3). They did not perceive changes in physical health (*M* = 4.0) and reported having experienced occasional boredom (*M* = 4.1) during lockdown. The means of subjective well-being indicators as well as the lockdown characteristics in the “Moderately positive” profile reflect the sample average.

### Languishing Profile

The “Languishing” profile reflects a state wherein individuals experienced more negative than positive feelings and reported not feeling good about their lives (see Fredrickson and Losada, 2005). Corresponding to this definition, participants with the “Languishing” profile reported negative experiences more often than the sample average (*M* = 4.7), whereas positive experiences were reported more rarely (*M* = 3.2) They also expressed ambivalence about their psychosocial well-being (*M* = 3.9). The “Languishing” profile contained 18% of participants, which made it the second largest profile. Similar to the “Moderately positive” profile, participants with this profile were also in lockdown with more than two people on average (*M* = 2.4; *x*^2^ = 1.360, *ns*.), reported a slight increase in childcare (*M* = 4.3 *x*^2^ = 3.214, *ns*.) and chores (*M* = 4.4; *x*^2^ = 1.856, *ns*.) as well as a decrease in workload (*M* = 2.7; *x*^2^ = 0.859, *ns*.). Different from the “Moderately positive” profile, participants with a “Languishing” profile reported more changes for the worse in their financial situation (*M* = 2.7; *x*^2^ = 12.505, *p* < 0.001) and physical health (*M* = 3.1; *x*^2^ = 16.226, *p* < 0.001). They also reported more frequent experiences of boredom (*M* = 5.3; *x*^2^ = 25.483, *p* < 0.001).

### Flourishing Profile

The “Flourishing” profile reflects a high level of subjective well-being that is indicated by frequent positive and infrequent negative experiences, as well as high levels of psychosocial well-being (Diener et al., 2010). Correspondingly, participants with this profile reported having positive experiences more often (*M* = 6.0) and negative experiences more rarely (*M* = 2.1) than participants with other profiles during lockdown. In addition, they reported higher levels of psychosocial wellbeing (*M* = 6.1) ~8% of participants fell into this profile type. In comparison to the “Moderately positive” profile, participants with the “Flourishing” profile were also in lockdown with others (*M* = 2.8; *x*^2^ = 0.215, *ns*.), reported a similar change in childcare (*M* = 4.9; *x*^2^ = 0.014, *ns*.) and chores (*M* = 4.9; *x*^2^ = 0.082, *ns*.), while perceiving a decrease in workload (*M* = 2.9; *x*^2^ = 0.004, *ns*.) and relatively unchanged physical health (*M* = 4.4; *x*^2^ = 2.740, *ns*.). Where the conditions of the “Flourishing” profile differ, however, is that that they did not perceive changes in their financial situation during lockdown (*M* = 4.0, *x*^2^ = 7.227, *p* = 0.007), whereas the “Moderately positive” perceived their financial situation had slightly deteriorated. Participants with the “Flourishing” profile also reported experiencing boredom very rarely, which was less frequently than those with a “Moderately positive” profile (*M* = 2.2, *x*^2^ = 60.922, *p* < 0.001).

### Mixed Feelings Profile

The “Mixed feelings” profile was applicable to 4% of the participants, who reported both positive and negative experiences equally often and more often than the average participant (*M* = 5.7 for positive and *M* = 5.4 for negative, respectively). Despite the high frequency of negative experiences, participants with the “Mixed feelings” profile reported higher than average psychosocial well-being (*M* = 6.0). Compared to the “Moderately positive” profile, those with the “Mixed feelings” profile did not differ to a significant extent in terms of changes in household chores (*M* = 4.4; *x*^2^ = 0.749, *ns*.). While they reported fewer changes in childcare responsibilities (*M* = 4.1; *x*^2^ = 2.941, *ns*.), workload (*M* = 3.8; *x*^2^ = 2.968, *ns*.), and their financial situation (*M* = 3.9; *x*^2^ = 2.016, *ns*.) than those with a “Moderately positive” profile, these differences were not statistically significant. What set the “Mixed feelings” profile apart from the “Moderately positive” profile was that they were in lockdown with more people (*M* = 3.3; *x*^2^ = 4.436, *p* = 0.035), experienced boredom more often (*M* = 5.4; *x*^2^ = 8.824, *p* = 0.003), and reported an increase in physical health (*M* = 5.2; *x*^2^ = 12.915, *p* < 0.001) during lockdown. These two characteristics also distinguished the “Mixed feelings” profile from the “Flourishing” profile (*x*^2^ = 4.171, *p* = 0.041 for change in physical health; *x*^2^ = 47.318, *p* < 0.001 for boredom).

### Apathetic

Finally, a small profile capturing 3% of the participants was identified, where members reported rarely having positive and negative experiences during lockdown (*M* = 2.0 for positive and *M* = 2.1 for negative, respectively). In addition, these profile members perceived lower than average psychosocial well-being (*M* = 3.1). We labeled this profile “Apathetic” to reflect the absence of affective experiences and their lower subjective well-being in general. Similar to the “Moderately positive” profile, participants with the “Apathetic” profile reported a slight decrease in workload (*M* = 3.0; *x*^2^ = 0.048, *ns*.) and their financial situation (*M* = 3.1; *x*^2^ = 0.368, *ns*.). They had less company in lockdown than those with a “Moderately positive” profile (*M* = 2.1; *x*^2^ = 4.229, *p* = 0.040). They also reported a decrease in household chores (*M* = 3.0, *x*^2^ = 5.877, *p* = 0.015) and childcare responsibilities (*M* = 3.5; *x*^2^ = 7.723, *p* = 0.005) compared to those with a “Moderately positive” profile. While they reported experiencing boredom during lockdown less often than those with a “Moderately positive” profile (*M* = 3.0; *x*^2^ = 5.736, *p* = 0.017), they also reported a stronger decrease in perceived physical health (*M* = 2.7; *x*^2^ = 10.785, *p* < 0.001). The low frequency of experienced boredom during the lockdown distinguishes the “Apathetic” profile from the “Languishing” profile (*M* = 4.1; *x*^2^ = 2.941, *ns*.), which is another profile that suffered during lockdown.

## Discussion

Our study displays distinct employee well-being profiles in the UK and France during the first coronavirus lockdown. The findings show that while a considerable group of employees was suffering during the lockdown, there were also those that were flourishing. Notably, perceived changes in financial situation and physical health as well as experienced boredom emerged as the prominent factors that distinguished these groups. These findings resonate with the loss and gain spirals postulated by Conservation of Resources Theory (COR; Hobfoll, 1989). Specifically, loss spirals describe a process potentially captured in the “Languishing” profile, wherein loss of valuable resources (e.g., money, security) leads to further loss (e.g., fitness) resulting in deteriorating well-being. In contrast, the “Flourishing” profile may involve a gain spirals process, where individuals are not facing loss (i.e., their financial situation had not deteriorated) and are therefore able to invest their time and energy in meaningful activity (i.e., experience less boredom), perhaps even more so given that less time was spent on work related activities.

Interestingly, the “Mixed feelings” profile illustrated a hybrid, wherein members reported frequent negative and positive experiences in combination with high psychosocial well-being during lockdown. This profile brings nuance to our understanding of subjective well-being as an absence of negative affective experiences (Danna and Griffin, 1999), especially during times of crisis where these experiences are likely to fluctuate at least for some individuals. While participants with the “Mixed feelings” profile reported boredom more often than members in other profiles, they also improved their physical health the most. It is possible that these profile members suffered from lack of stimuli during lockdown, but also had the means (e.g., exercise; Lades et al., 2020) to successfully cope with it and other stresses of the pandemic, which enabled them to sustain high subjective well-being. In addition, these employees had bigger households, which may have contributed to stronger social networks that have been shown to maintain subjective well-being amidst the pandemic (Jaspal and Breakwell, 2020). Perceiving social support from others may have enabled them to build resilience (Cooke et al., 2019), which can protect employee well-being from COVID-19 related stresses (Paredes et al., 2020). Participants with the “Mixed feelings” may have thus been able to sustain their well-being by increasing their resources (i.e., physical health) even during dreary times, which aligns with the Gain paradox principle of COR theory (Hobfoll et al., 2018).

These findings notably complement but also problematize some prior studies that signal contrasting well-being impacts of the Covid-19 pandemic based on socioeconomic factors such as income and education (Wanberg et al., 2020), or occupation (Recchi et al., 2020), or even precarious/vulnerable positions (Apouey et al., 2020; Enriquez and Goldstein, 2020; Jaspal and Breakwell, 2020; Wang et al., 2020). The fact that we did not find any one socioeconomic or occupational factor to predict profile membership implies that, at least in this type of crisis, employee well-being is likely to differ also within and not just across socioeconomic groups.

Notably, rather than socioeconomic factors, our findings highlight the role of *changes* in resources, such as household financial situation and physical health, in distinguishing different employee well-being profiles. While more affluent employees may be better able to protect their well-being from the adverse effects of crises, our study aligns with the central tenet of COR theory that emphasizes *loss* of valued resources (e.g., money, health, security) as the main threat to well-being (Hobfoll, 1989). This key finding can prove helpful for research seeking to understand socioeconomic influences of well-being in a more nuanced way. Furthermore, nationality was not a predictor of profiles, which implies that the profiles were not country specific.

### Limitations and Suggestions for Further Research

This study has some limitations. First, the use of cross-sectional self-report data involves possible method bias (Podsakoff et al., 2012). Additionally, it is not possible to assess whether well-being actually changed for these people during lockdown. For example, it may be that the “Apathetic” profile reflects a chronic condition (e.g., depression), the origins of which may extend to before the COVID-19 era. Nevertheless, the methodological approach enabled us to highlight nuances in employee well-being during a unique point in time. Future studies could test whether similar profiles can be replicated at a later stage in the pandemic, and follow-up on how they evolve over time. Pre-pandemic era studies have shown that employee well-being tends to remain stable over time (Seppälä et al., 2015), and that employees in favorable well-being profiles in particular tend to maintain and even increase their resources (Mäkikangas et al., 2016). It is not yet clear whether these findings can be applied to the conditions of the prolonged pandemic. For example, examining whether new profiles emerge after a series of lockdowns might add to our understanding of employee well-being during the pandemic. Furthermore, studying whether and why employees in the “Flourishing” profile are able to retain their well-being after a long period of restricted life could reveal important insights on resilience to stress and adversity.

Second, it is worth mentioning that in order to ensure that results could be interpreted similarly across UK and French participants (i.e., to achieve measurement invariance), we needed to remove one item reflecting psychosocial well-being and another reflecting negative experiences. While we deemed that these constructs were still sufficiently reflected by the remaining items (Cheung and Rensvold, 1999), future studies seeking to replicate this study should note these adjustments made to the measurement instruments. Nevertheless, we believe that future research utilizing the subjective well-being measure by Diener et al. (2009) in cross-country samples can benefit from the information concerning differences in these two items across cultural contexts and study them further.

Third, while closer examination of the changes and experiences that may underlie employee well-being during lockdown is beyond the scope of our study, we believe that exploring these characteristics further provides interesting areas for future research. For example, our study implied that deterioration of the household's financial situation was a distinctive characteristic of the “Languishing” profile. While we did not examine the reasons for these events, it is likely that for many employees these financial changes relate to changes in their (or their spouse's) employment situation or their opportunities to work. The ongoing health crisis is likely to continue causing this type of job insecurity for many employees whose jobs are heavily impacted by the pandemic (e.g., hospitality industries, restaurants, gyms), which may further deteriorate their health and well-being (De Witte, 1999). Previous studies have shown that a sense of control (e.g., Vander Elst et al., 2014) and employability (De Cuyper et al., 2008) may mitigate the negative effects of job insecurity on employee well-being. Hence, future studies could investigate whether and how organizations can leverage these resources to support their employees in navigating these uncertain times with their well-being intact. Moreover, whether employees flourished because they could spend more time teleworking from home and spend more time with their families, or whether the employees in the “Mixed feelings” profile were able to cope with boredom and manage their well-being during lockdown by exercising more represent the types of questions that can pave the way to potentially valuable insights in the post-COVID-19 era where we are likely to see an increase in remote work.

## Data Availability Statement

The raw data supporting the conclusions of this article will be made available by the authors, without undue reservation.

## Ethics Statement

Ethical review and approval was not required for the study on human participants in accordance with the local legislation and institutional requirements. Written informed consent for participation was not required for this study in accordance with the national legislation and the institutional requirements.

## Author Contributions

All authors listed have made a substantial, direct and intellectual contribution to the work, and approved it for publication.

## Conflict of Interest

The authors declare that the research was conducted in the absence of any commercial or financial relationships that could be construed as a potential conflict of interest.
