# Supplementary material for: Employee Well-Being Profiles During COVID-19 Lockdown: A Latent Profile Analysis of French and UK Employees
Source: Front Psychol. 2021 Jun 9;12:645300. doi: 10.3389/fpsyg.2021.645300 (PMC8219910; doi:10.3389/fpsyg.2021.645300)
Supplement: Supplementary file 1 [file Table_1.pdf]

Table 4. Three-Step results for socioeconomic and occupational antecedents of well-being profiles (N=652)

	Flourishing	Languishing	Mixed feelings	Apathetic
	β(SE)	*β (SE)*	*β (SE)*	*β (SE)*
Age	.216(.38)	-.103(.31)	-.421(.51)	-.213(.57)
Gender (0=Male 1=Female)	.335(.39)	.221(.31)	-.493(.60)	-.556(.67)
Low income	-.242(.43)	.300(.32)	.112(.55)	.714(.61)
Higher education	-.470(.42)	.182(.33)	.728(.83)	.428(.70)
Country (0=France 1=UK)	-.314(.38)	-.286(.31)	-.009(.51)	-.064(.56)
Working remotely	.074(.38)	-.245(.33)	.627(.67)	-.333(.74)
Professional / Managerial position	.448(.50)	-.127(.47)	.208(.70)	.996(.75)

***p < .001 **p < .01 *p < .05

Note. The “Moderately positive” profile is the referent profile. Positive coefficient values indicate that higher values of the antecedent render an individual more likely to be in the target profile than in the referent profile. Negative coefficient indicates that a higher value in the antecedent renders one more likely to belong to the referent profile.
